# Multilevel barriers to clinical and nutritional research in Latin America: a socioeconomic comparative analysis

**DOI:** 10.3389/fnut.2025.1599344

**Published:** 2025-12-31

**Authors:** Evelyn Frias-Toral, Jaime Angamarca-Iguago, Isabel Calvo Higuera, Jorge Carriel-Mancilla, Guillermo Contreras, Raquel Franco-Nuñez, Rosa Larreategui Arosemena, Claudia Maza Moscoso, Javier Restrepo, Jaen Cagua-Ordoñez, Alison Simancas-Racines, Claudia Reytor-González, Daniel Simancas-Racines

**Affiliations:** 1 Escuela de Medicina, Universidad Espíritu Santo, Samborondón, Ecuador; 2 Division of Research, Texas State University, San Marcos, TX, United States; 3 Universidad UTE, Facultad de Ciencias de la Salud Eugenio Espejo, Centro de Investigación en Salud Pública y Epidemiología Clínica (CISPEC), Quito, Ecuador; 4 Escuela de Medicina, Pontificia Universidad Católica del Ecuador, Santo Domingo, Ecuador; 5 Hospital General de Tijuana, Tijuana, Mexico; 6 School of Medicine, Universidad Católica Santiago de Guayaquil, Guayaquil, Ecuador; 7 Departamento de Emergencia, Hospital Guillermo Almenara, Lima, Peru; 8 Universidad San Ignacio de Loyola, Lima, Peru; 9 Facultad de Ciencias Médicas, Hospital de Clínicas, Departamento de Nutrición, Universidad Nacional de Asunción, San Lorenzo, Paraguay; 10 Hospital Quirúrgico de Alta Complejidad de Ciudad de la Salud de la Caja de Seguro Social de Panamá, Ciudad de Panamá, Panama; 11 Division of Education and Research, Centro Médico Militar, Guatemala City, Guatemala; 12 Research and Development Pharmaceutical Products, World Health S.A.S Center, Poblado, Medellín, Colombia; 13 Universidad Técnica de Cotopaxi, Facultad de Ciencias Agropecuarias y Recursos Naturales, Carrera de Agropecuaria, Latacunga, Ecuador; 14 Facultad de Salud y Bienestar, Pontificia Universidad Católica del Ecuador, Quito, Ecuador

**Keywords:** clinical research barriers, global health equity, health research capacity, knowledge partnership, Latin America, nutrition science, research inequality, scientific infrastructure

## Abstract

**Introduction:**

Clinical and nutritional research in Latin America faces significant challenges that limit scientific development and evidence-based healthcare. Understanding these barriers is essential for developing effective strategies to enhance research capacity in the region. This study aimed to identify multilevel barriers to clinical and nutritional research in Latin America and compare them between countries of different socioeconomic levels.

**Methods:**

A cross-sectional study was conducted with 327 healthcare professionals involved in clinical and nutritional research across Latin America. Data collection occurred via an online survey in which participants rated the importance of 16 potential barriers on a 3-point Likert scale. Analysis included descriptive statistics, chi-square tests to compare barriers between upper-middle and lower-middle-income countries, logistic regression to identify predictors of research participation, and k-means cluster analysis to identify researcher profiles.

**Results:**

Funding (84.4%), research materials (71.6%), and time constraints (70.9%) emerged as the most significant barriers across all countries. Three barriers showed statistically significant differences between income levels: participant commitment (73.6% vs. 42.6%, *p* < 0.001), frequent appointments (56.6% vs. 37.8%, *p* = 0.02), and language barriers (39.6% vs. 22.9%, *p* = 0.02), all of which were higher in lower-middle-income countries. Logistic regression identified the importance of research materials (OR = 0.36, *p* = 0.002) and telemedicine (OR = 1.74, *p* = 0.044) as significant predictors of research participation. Cluster analysis revealed three distinct researcher profiles based on barrier perception patterns.

**Conclusion:**

Multilevel barriers to research in Latin America are dominated by universal resource constraints (funding, materials, time), with lower-middle-income countries facing additional challenges in participant engagement and study logistics. The relative homogeneity of most barriers across income groups suggests that regional and institutional factors may be more influential than national income levels. These findings provide a foundation for developing targeted strategies to strengthen research capacity and infrastructure across Latin America.

## 1 Introduction

Clinical and nutritional research plays a pivotal role in tackling global health challenges, especially in low- and middle-income countries (LMICs), where the burden of disease is disproportionately high. Yet, these settings face numerous obstacles, including limited funding, inadequate infrastructure, and complex regulatory environments that collectively impede the development of evidence-based interventions (1, 2).

In Latin America, such barriers are further exacerbated by pronounced health disparities and limited resources, which hinder the development of effective public health policies. Regions characterized by socioeconomic inequality face persistent gaps in healthcare access and quality. Therefore, it is essential to implement health system reforms that promote universality, comprehensiveness, and sustainability—underscoring the urgent need to strengthen research capacity (3).

At the same time, global shifts in the food system and ongoing nutrition transitions, characterized by rapid urbanization, changing dietary patterns, and a rising prevalence of ultra-processed foods, have driven increases in non-communicable diseases such as obesity, diabetes, and cardiovascular disease (4–13). These trends have given rise to the “double burden” of malnutrition, wherein undernutrition and overnutrition coexist within the same populations, further complicating public health responses (14–16).

Moreover, systemic constraints, including insufficient training, a dearth of interdisciplinary collaboration, and weak regulatory frameworks, continue to limit the capacity to conduct high-quality research in resource-constrained environments. In this context, pragmatic clinical trials designed to test interventions under real-world conditions offer an appealing strategy for generating actionable evidence. Yet, their successful implementation requires overcoming both operational hurdles and regulatory challenges (2, 3).

Against this backdrop, the intersection of clinical and nutritional research in Latin America presents both unique challenges and promising opportunities. Given the region’s diverse cultural, economic, and epidemiological landscapes, tailored research approaches are essential to address local priorities. This study aims to identify and analyze multilevel barriers to clinical and nutritional research in Latin America, with a focus on understanding differences across countries with varying socioeconomic levels. By providing a comprehensive assessment of these barriers, the study seeks to inform strategies to strengthen research capacity and foster equitable health outcomes across the region.

## 2 Methodology

### 2.1 Study design and setting

This cross-sectional study employed a quantitative approach to identify and analyze barriers to clinical and nutritional research in Latin America and the Caribbean. The study was conducted between September 2021 and February 2022. All participants provided informed consent before completing the survey.

### 2.2 Participant selection and recruitment

Healthcare professionals involved in clinical and nutritional research across Latin America and the Caribbean were invited to participate through a multi-stage recruitment process. Initial recruitment was conducted through purposive sampling, with outreach to research institutions, universities, and hospitals across the region. Primary recruitment channels included professional societies affiliated with FELANPE (Latin American Federation of Parenteral and Enteral Nutrition) and ILAS (Latin American Institute of Food Science), institutional email lists, newsletters, and social media platforms (e.g., LinkedIn, ResearchGate). The survey link was distributed through these channels along with a standardized invitation letter explaining the study’s purpose and procedures. Subsequently, snowball sampling was employed, wherein initial participants were encouraged to share the survey link with eligible colleagues in their professional networks. Participants accessed the questionnaire through a secure Google Forms link that required informed consent before proceeding to the survey questions. The survey was accessible via computer, tablet, or smartphone to maximize participation across different technological contexts.

#### 2.2.1 Inclusion criteria

Inclusion criteria: (1) being a healthcare professional (including physicians, nutritionists/dietitians, pharmacists, nurses, and related fields); (2) having participated in or currently being involved in clinical or nutritional research; and (3) practicing in a Latin American or Caribbean country at the time of survey completion.

#### 2.2.2 Exclusion criteria

Exclusion criteria, defined independently from inclusion criteria, included: (1) incomplete survey responses (less than 80% completion rate); (2) duplicate submissions (verified by IP address and timestamps); (3) lack of informed consent; (4) respondents who were not healthcare professionals; and (5) surveys completed in less than 5 min, suggesting insufficient attention to responses.

### 2.3 Ethical consideration

This study was classified as exempt from formal ethics committee review because it involved an anonymous, minimal-risk survey of healthcare professionals without patient data or interventions, in accordance with international guidelines, including the Council for International Organizations of Medical Sciences (CIOMS) International Ethical Guidelines for Health-Related Research Involving Humans and the U.S. Common Rule 45 CFR 46.104 (17, 18). All participants provided explicit informed consent electronically before accessing the survey questions. No personally identifiable information was collected, ensuring participant anonymity and confidentiality.

### 2.4 Survey development and validation

The survey instrument was developed based on a comprehensive literature review of barriers to clinical and nutritional research in low- and middle-income countries, combined with expert panel consultation. The initial questionnaire was evaluated for face and content validity by a panel of six experts in clinical and nutritional research from across the region, representing diverse disciplinary backgrounds and institutional settings.

The survey was then pilot-tested with 20 researchers not included in the final sample to assess clarity, comprehensibility, and time required for completion. Based on feedback from the pilot test, modifications were made to improve question wording, response option clarity, and survey flow. Internal consistency reliability was assessed using Cronbach’s alpha for the barrier perception scale, yielding an acceptable value (*α* = 0.78). Test–retest reliability was evaluated with a subset of 15 participants who completed the survey twice with a two-week interval, demonstrating good temporal stability (intraclass correlation coefficient [ICC] = 0.82, 95% CI: 0.61–0.93). These reliability measures confirm the consistency and stability of our data collection instrument.

The final questionnaire consisted of three sections.

#### 2.4.1 Section 1: demographic and professional information

This section included questions on sex (male/female), age group (categorized as: ≤25 years, 26–35 years, 36–45 years, 46–55 years, 56–65 years, >65 years), profession (nursing, pharmacy, medicine, nutrition/dietetics, other), highest education level attained (doctorate/postdoctorate, specialty/subspecialty, master’s degree, bachelor’s degree, other), country of residence, years of clinical experience (1–3 years, 4–6 years, 7–9 years, >10 years), and institutional affiliation (university, teaching hospital, research center, non-teaching hospital, other).

#### 2.4.2 Section 2: research experience

This section assessed participants’ involvement in research protocols, including current or past participation in research projects (yes/no), types of research conducted (qualitative studies, observational studies, clinical trials, post-marketing studies, other), number of research projects participated in (1–5, 6–10, >10, none), and institutional research approval processes.

#### 2.4.3 Section 3: barriers to research

Participants rated the importance of 16 potential barriers to clinical and nutritional research using a 3-point Likert scale (1 = high importance, 2 = medium importance, 3 = low importance). The barriers assessed included: (1) funding limitations; (2) research materials (medicines, reagents, equipment); (3) time constraints due to workload; (4) ethics committee procedures; (5) competent authority/regulatory procedures; (6) trained personnel availability; (7) additional biopsies required; (8) insurance/coverage issues; (9) participant commitment to study protocols; (10) risks associated with the intervention; (11) laboratory test requirements; (12) frequent appointments/follow-up visits; (13) strict inclusion/exclusion criteria; (14) language barriers; (15) patient enrollment difficulties; and (16) telemedicine utilization for follow-up.

The decision to use a 3-point Likert scale rather than the more common 5- or 7-point scales was deliberate and based on several methodological considerations. First, research has shown that simpler response scales can reduce respondent burden and cognitive load, particularly in international surveys where respondents may have varying levels of language proficiency. Second, the 3-point scale (high, medium, low importance) provides sufficient discrimination for practical decision-making while minimizing ambiguity in the middle categories that often plague longer scales. Third, pilot testing indicated that participants found the 3-point scale intuitive and easier to complete consistently across all 16 barrier items. Finally, the scale retained adequate psychometric properties as evidenced by acceptable internal consistency (*α* = 0.78) and test–retest reliability (ICC = 0.82).

The survey was made available in Spanish, Portuguese, and English to accommodate participants from different countries in the region. Professional translators ensured linguistic equivalence across all three versions, with back-translation procedures implemented to verify accuracy.

### 2.5 Income classification methodology

Countries were classified according to the World Bank income categories for fiscal year 2022, which corresponds to the survey period from September 2021 to February 2022. The World Bank categorizes economies based on gross national income (GNI) per capita, calculated using the World Bank Atlas method. For fiscal year 2022, the income groups were defined as follows: low-income economies (GNI per capita ≤$1,085), lower-middle-income economies ($1,086–$4,255), upper-middle-income economies ($4,256–$13,205), and high-income economies (≥$13,206).

#### 2.5.1 Country-specific classifications

The following countries represented in our sample were classified according to official World Bank designations: High-income: Chile, Spain, United States, Panama, Uruguay (*n* = 19, 5.8% of the sample). Upper-middle-income: Argentina, Brazil, Colombia, Costa Rica, Ecuador, Mexico, Peru, Dominican Republic (*n* = 249, 76.1% of sample). Lower-middle-income: El Salvador, Guatemala, Nicaragua, Paraguay (*n* = 53, 16.2% of sample).

#### 2.5.2 Treatment of unclassified countries

Two countries in our sample presented classification challenges. Cuba (*n* = 5) lacks a recent official World Bank income classification because standardized economic data are unavailable. Venezuela (*n* = 1), which has historically been classified as upper-middle-income, has experienced a severe economic crisis since 2014, and current World Bank data are incomplete. To maintain methodological rigor and avoid arbitrary assumptions about income status in the absence of reliable data, both Cuba and Venezuela were classified as “Unclassified” (total *n* = 6, 1.8% of sample) and excluded from inferential statistical comparisons. Their descriptive statistics are presented separately, and this classification limitation is explicitly acknowledged.

### 2.6 Sample size considerations and statistical power

Given the use of purposive and snowball sampling techniques, traditional priori power calculations were not applicable for this study design. These non-probabilistic sampling methods do not allow conventional sample size estimation based on population parameters, as the sampling frame and population denominator cannot be precisely defined. Instead, sample size considerations were guided by practical and methodological factors: (1) achieving representation across multiple Latin American and Caribbean countries; (2) ensuring adequate sample sizes for subgroup analyses by income level and professional categories; (3) meeting minimum requirements for multivariate statistical analyses, particularly logistic regression (minimum 10 events per predictor variable) and (4) considering resource constraints and feasibility of recruitment through professional networks (2).

#### 2.6.1 Minimum sample size threshold for statistical testing

Following established guidelines for group comparisons in exploratory research (19). We prespecified a minimum threshold of *n* ≥ 30 participants per group for conducting inferential statistical tests. This threshold is based on the Central Limit Theorem, which suggests that sampling distributions approximate normality when *n* ≥ 30, and on practical considerations for achieving adequate statistical power (typically ≥80%) for detecting medium-to-large effect sizes in comparative analyses. Groups with fewer than 30 participants were analyzed descriptively only, without statistical hypothesis testing, to avoid Type I and Type II errors associated with underpowered analyses.

Based on similar cross-sectional studies in healthcare research and recommendations for exploratory research in low- and middle-income countries, we aimed to target a sample of 300–350 participants to provide sufficient heterogeneity for meaningful comparisons while maintaining analytical robustness. The final sample of 327 participants provided stable estimates for our planned analyses and adequate representation across the region.

#### 2.6.2 Limitations of non-probabilistic sampling

We acknowledge that the non-probabilistic sampling approach limits generalizability to the broader population of healthcare researchers in Latin America. This study employed purposive and snowball sampling as an exploratory approach, consistent with research capacity studies in LMICs, where probabilistic sampling is often impractical (20). As such, our findings are hypothesis-generating and not intended for population-level inference. Results should be interpreted as representing the experiences and perspectives of healthcare professionals engaged in clinical and nutritional research who were accessible through our recruitment networks, rather than a statistically representative sample of all such professionals in the region.

Data collection occurred between September 2021 and February 2022 through an online Google Forms survey. The questionnaire took approximately 15–20 min to complete. Three follow-up reminders were sent at two-week intervals to maximize response rate, without being overly intrusive. Participants were explicitly requested to complete the survey only once, and both IP address and timestamp verification were used to identify potential duplicate submissions. All responses were automatically recorded in a secure, password-protected database accessible only to the research team.

### 2.7 Missing data management

Missing data were systematically assessed and managed throughout the study. Surveys with completion rates below 80% were excluded as per our predefined criteria (*n* = 43 surveys excluded on this basis). In the final sample of 327 participants, the overall missing data rate was very low (0%) because the Google Forms platform was configured to require responses to all key questions before submission. This complete-case dataset eliminated the need for imputation procedures and the associated assumptions about missing-data mechanisms.

### 2.8 Variable definitions and measurements

#### 2.8.1 Outcome variable

The primary outcome variable was current or past participation in research protocols, assessed with the question: “Are you currently participating or have you participated in any research protocol?” with dichotomous response options (1 = Yes, 2 = No).

#### 2.8.2 Predictor variables

Income group: Based on World Bank classifications as described above, countries were grouped for the primary analysis as follows: Main inferential analysis: Upper-middle-income vs. Lower-middle-income (*n* = 302 total). Descriptive only: High-income (*n* = 19, excluded due to *n* < 30 threshold). Descriptive only: Unclassified (*n* = 6, excluded due to lack of valid income classification and *n* < 30).

Demographics: Sex (male/female), age (ordinal categories as described above), education level (ordinal scale from bachelor’s degree to doctorate), profession (categorical: nursing, pharmacy, medicine, nutrition/dietetics, other), clinical experience (ordinal: 1–3, 4–6, 7–9, >10 years).

Barriers: Each of the 16 barriers was measured on the 3-point Likert scale described above. For primary analyses, barriers were dichotomized as “high importance” (score = 1) versus “medium/low importance” (scores = 2 or 3) to facilitate interpretation and enable chi-square testing. Sensitivity analyses using the original ordinal scale and nonparametric tests (Mann–Whitney U) were conducted to verify the robustness of the findings.

### 2.9 Statistical analysis

All statistical analyses were performed using Python (version 3.9) with the following libraries: pandas (version 1.4.2) for data manipulation, numpy (version 1.22.3) for numerical operations, scipy (version 1.8.0) for statistical tests, statsmodels (version 0.13.2) for regression modeling, and scikit-learn (version 1.0.2) for cluster analysis. Statistical significance was set at *p* < 0.05 for all analyses, and all tests were two-tailed. All analysis code is provided as Supplementary materials to ensure full reproducibility.

#### 2.9.1 Descriptive statistics

Descriptive statistics were calculated for all variables. Categorical variables were presented as frequencies and percentages. Continuous variables were described using means and standard deviations when normally distributed, or medians and interquartile ranges (IQR) for skewed distributions. Barrier importance ratings were ranked by the percentage of participants who rated each barrier as having “high importance” overall and by income group.

#### 2.9.2 Main comparative analysis: upper-middle vs. lower-middle income

The primary inferential statistical comparisons were conducted between upper-middle-income and lower-middle-income countries, as these were the only income groups meeting the *n* ≥ 30 threshold (*n* = 249 and *n* = 53, respectively; total *n* = 302). For categorical variables (including dichotomized barriers): Chi-square tests of independence were used when all expected cell frequencies were ≥5. When expected frequencies were <5, Fisher’s exact test was used instead, as it provides exact *p*-values without relying on asymptotic approximations. The choice of test for each variable was determined by examining the expected frequencies in the contingency tables. For ordinal variables (original 3-point Likert scales): Mann–Whitney U tests were conducted as sensitivity analyses to verify that dichotomization did not materially affect conclusions. The Mann–Whitney U test is a nonparametric alternative to the independent-samples t-test that does not assume normality and is appropriate for ordinal data. Effect sizes: For significant chi-square results, Cramér’s V was calculated to quantify effect size (small: 0.10, medium: 0.30, large: 0.50). For Mann–Whitney U tests, rank-biserial correlation was computed as an effect size measure.

#### 2.9.3 High-income and unclassified groups: descriptive presentation only

For high-income countries (*n* = 19) and unclassified countries (*n* = 6), only descriptive statistics (frequencies, percentages, medians, IQRs) were calculated and reported. “No statistical hypothesis tests were performed” for these groups due to insufficient sample sizes (*n* < 30), which would result in low statistical power and unreliable *p*-values. All tables and figures clearly indicated which results were descriptive only versus those that were statistically tested, maintaining transparency about the scope of inference.

#### 2.9.4 Logistic regression analysis

Multivariable logistic regression was performed to identify independent predictors of research protocol participation among the 302 participants in the main analysis sample (from upper-middle- and lower-middle-income countries). The binary outcome was coded as 1 = currently participating or have participated, 0 = never participated.

Model building strategy: We employed a theory-driven approach combined with bivariate screening. Variables were included in the multivariable model if they: (1) showed associations with the outcome in bivariate analyses at *p* < 0.20; (2) were identified as theoretically necessary based on prior literature; or (3) were demographic confounders (age, sex, education). To avoid overfitting, we adhered to the rule of thumb of at least 10 events (participations) per predictor variable in the model, limiting our model to approximately 17–20 predictors given 177 participation events.

Model specifications: The final model included: income group (upper-middle vs. lower-middle), sex (male vs. female), age (ordinal), education level (ordinal), profession (categorical, with nutrition/dietetics as reference), and key barriers identified through bivariate screening (funding, research materials, time constraints, patient enrollment, telemedicine; each dichotomized as high vs. medium/low importance).

Model diagnostics: Multicollinearity was assessed using variance inflation factors (VIF), with VIF < 5 considered acceptable and VIF < 3 ideal (14). Model fit was evaluated using the Hosmer-Lemeshow goodness-of-fit test (*p* > 0.05 indicates acceptable fit)—Discrimination ability was assessed using the area under the receiver operating characteristic curve (AUC-ROC), with AUC > 0.70 considered acceptable. Pseudo-R^2^ (Nagelkerke) was calculated to estimate the proportion of variance explained. Residual analysis and Cook’s distance were examined to identify influential outliers.

Reporting: Odds ratios (OR) with 95% confidence intervals (CI) and *p*-values were reported for all predictors. ORs > 1 indicate increased odds of research participation; ORs < 1 indicate decreased odds.

#### 2.9.5 Cluster analysis

K-means clustering was conducted to identify distinct groups of researchers based on their patterns of barrier perception across all 16 barriers. Before clustering, barrier scores were standardized (z-scores) to ensure equal weighting of all variables.

Determination of optimal number of clusters: Multiple methods were used in conjunction: 1. Elbow method: plotting within-cluster sum of squares (inertia) against number of clusters. 2. Silhouette coefficient: assessing cluster cohesion and separation (range: −1 to +1, higher is better). 3. Interpretability: ensuring clusters had meaningful and distinct profiles. Based on these criteria, k = 3 was selected as optimal, balancing statistical metrics with practical interpretability.

Methodological note on k-means for ordinal data: We acknowledge that k-means clustering assumes continuous variables and uses Euclidean distance, which may not be ideally suited for ordinal 3-point Likert data. However, several considerations supported this choice: (1) the ordinal scale approximates interval properties given relatively balanced response distributions; (2) k-means provides interpretable centroid-based clusters; (3) comparative analyses using alternative methods (hierarchical clustering with Ward’s linkage, k-modes for categorical data) yielded substantively similar cluster structures, supporting robustness; and (4) this approach maintains consistency with the original manuscript framework while acknowledging its limitations.

Cluster characterization: Mean barrier scores for each cluster were calculated and ranked to identify the top barriers (lowest mean scores = highest perceived importance) characterizing each cluster. Demographic and professional characteristics of cluster members were also described.

#### 2.9.6 Sensitivity analyses

To assess the robustness of findings and address reviewer concerns about alternative grouping strategies, we conducted the following pre-specified sensitivity analyses:

Sensitivity Analysis 1: Upper-middle vs. Lower-middle + Unclassified (excluding High-income). This analysis added the six unclassified participants (Cuba, Venezuela) to the lower-middle-income group, creating a more inclusive “lower resource” category while still excluding high-income countries. Chi-square tests were re-run for all barriers.

Sensitivity Analysis 2: Upper-middle + High vs. Lower-middle + Unclassified. This analysis combined upper-middle- and high-income countries into a “higher resource” group and lower-middle and unclassified countries into a “lower resource” group, maximizing sample sizes for both groups (*n* = 268 vs. *n* = 59). This represents the most inclusive 2-group comparison possible with the available data.

Sensitivity Analysis 3: Mann–Whitney U tests on original Likert scales. As described above, all comparisons were repeated using non-parametric tests on the original 3-point ordinal scales to verify that dichotomization did not alter substantive conclusions.

Sensitivity Analysis 4: Logistic regression with alternative specifications. The logistic regression model was re-estimated with alternative variable combinations to assess the stability of coefficient estimates and significance levels.

### 2.10 Statement on generalizability

The findings of this study should be interpreted in the context of the sampling methodology employed. While every effort was made to recruit a diverse sample across multiple countries and professional backgrounds, the use of non-probabilistic sampling (purposive and snowball) means that results are not statistically generalizable to all healthcare professionals conducting clinical and nutritional research in Latin America. Instead, findings represent the experiences and perspectives of research-engaged professionals accessible through our professional networks (FELANPE, ILAS, and affiliated institutions). The study provides valuable hypothesis-generating insights and identifies barriers warranting further investigation through representative sampling designs.

Moreover, the marked imbalance in sample sizes across income groups (upper-middle: *n* = 249, 76%; lower-middle: *n* = 53, 16%; high: *n* = 19, 6%; unclassified: *n* = 6, 2%) limits the scope of statistical inference. The main comparative analyses were restricted to upper-middle-income versus lower-middle-income countries, as these were the only groups meeting the *n* ≥ 30 threshold. Findings for high-income and unclassified countries are descriptive only and should be interpreted with caution.

## 3 Results

### 3.1 Demographic characteristics

A total of 327 healthcare professionals from 19 Latin American and Caribbean countries completed the survey between September 2021 and February 2022. Most respondents were from upper-middle-income countries (*n* = 249; 76.1%), followed by lower-middle-income countries (*n* = 53; 16.2%). Brazil (27.2%), Mexico (14.1%), and Guatemala (11.3%) contributed the most significant numbers of participants, while eight countries had fewer than 10 respondents, indicating some geographical concentration in the sample. The geographic and income-level distribution is presented in Supplementary Figure S1, including the pre-specified threshold of *n* ≥ 30 used to determine eligibility for inferential analyses.

Based on the predefined threshold of *n* ≥ 30 for inferential analyses, only upper-middle- and lower-middle-income groups met the minimum sample size requirements (combined *n* = 302; 92.4% of the total). Accordingly, statistical comparisons were restricted to these groups. Participants from high-income countries (*n* = 19) and unclassified countries (*n* = 6) were included descriptively only due to insufficient sample size.

The sample was predominantly female (73.1%), with the largest age groups being 36–45 years (30.9%), 26–35 years (24.8%), and 46–55 years (22.0%). Nutritionists/dietitians represented the most common profession (49.8%), followed by physicians (31.5%), pharmacists (9.2%), and nurses (7.3%). Regarding education, 44.6% held a specialty or subspecialty degree, 27.2% held a master’s degree, and 28.2% reported other academic training. Most participants had more than 10 years of clinical experience (73.4%), and 61.2% reported current or prior involvement in research protocols. Full demographic details are provided in Table 1.

**Table 1:** Participant demographics.

Characteristic	Category	*n* (%)
Sex	Female	239 (73.1)
	Male	88 (26.9)
Age (years)	36–45	101 (30.9)
	46–55	72 (22.0)
	26–35	81 (24.8)
	Others	73 (22.3)
Profession	Nutrition/dietetics	163 (49.8)
	Physician	103 (31.5)
	Pharmacist	30 (9.2)
	Nurse	24 (7.3)
	Others	7 (2.1)
Education level	Master’s degree	89 (27.2)
	Specialty/subspecialty	146 (44.6)
	Others	92 (28.2)
Clinical experience	>10 years	240 (73.4)
	Others	87 (26.6)

Demographic patterns were similar between upper-middle- and lower-middle-income countries, including sex distribution, age structure, professional background, and research participation (58.2% vs. 60.4%; *p* = 0.76). High-income and unclassified groups showed extreme values—for example, research participation rates of 89.5 and 100%, respectively—although these findings should be interpreted cautiously given their very small sample sizes.

### 3.2 Barriers to clinical and nutritional research

**Figure 1:**
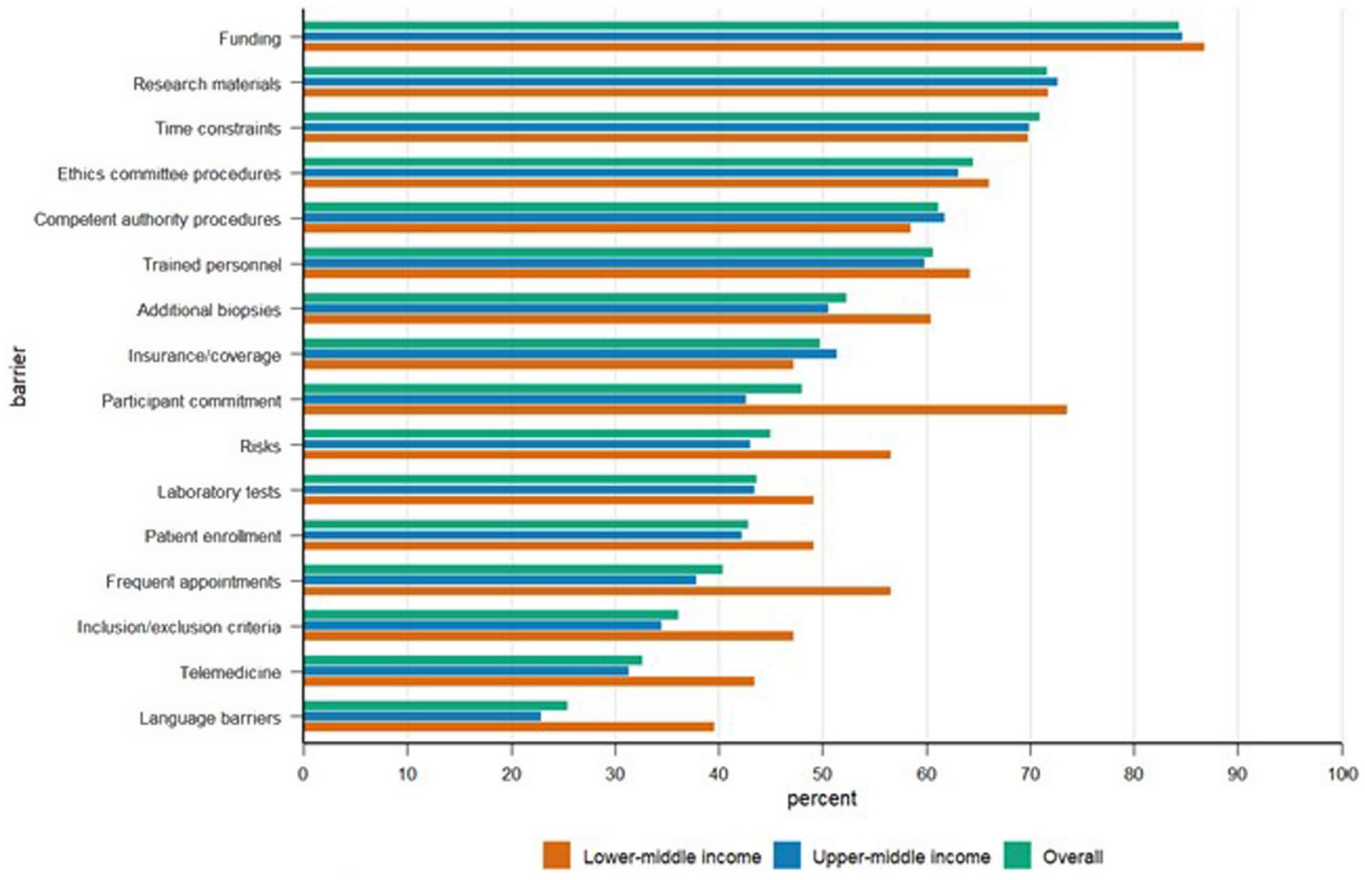
High-importance research barriers by country income group. Percentage of respondents rating each barrier as high importance, stratified by country income group. Data represent the proportion of participants from lower-middle income (*n* = 53), upper-middle income (*n* = 249), and the overall combined sample (*n* = 302) who classified each barrier as highly important. Barriers are ordered from highest to lowest overall importance.

Among the 327 respondents, barriers were ranked by the proportion rating each as “high importance.” Three obstacles consistently emerged as the most critical: limited funding (84.4%), insufficient research materials such as reagents, medicines, and equipment (71.6%), and time constraints resulting from clinical workload (70.9%). These were followed by ethics committee procedures (64.5%), competent authority or regulatory procedures (61.2%), and shortages of trained personnel (60.6%). Detailed rankings are provided in Table 2, and full distributions are shown in Figure 1.

**Table 2:** Top barriers to clinical and nutritional research (validated).

Barrier	High importance (%)
Funding	84.4
Research materials	71.6
Time constraints	70.9
Ethics committee procedures	64.5
Competent authority procedures	61.2
Trained personnel	60.6
Additional biopsies	52.3
Insurance/coverage	49.8
Participant commitment	48.0
Risks	45.0

Moderate-frequency barriers—including additional biopsies, insurance or coverage issues, participant commitment, and perceived risks—were each rated as highly important by roughly half of respondents. Less frequently cited obstacles included challenges with laboratory tests, patient enrollment, frequent appointments, inclusion and exclusion criteria, telemedicine, and language barriers, indicating comparatively lower perceived importance for most participants.

#### 3.2.1 Barrier profiles by country income level

Descriptive patterns across income groups closely aligned with the overall results. In upper-middle-income countries, the three leading barriers were identical to the full sample—funding, research materials, and time constraints—followed by ethics and regulatory procedures. In lower-middle-income countries, funding remained the top barrier; however, participant commitment ranked notably higher than in upper-middle-income settings (73.6% vs. 42.6%). Other frequently cited barriers in this group included research materials, time constraints, ethics committee procedures, and limited trained personnel. Respondents from high-income countries, presented descriptively due to small sample size, placed strong emphasis on time constraints (94.7%), while patient enrollment appeared less problematic than in other groups. Results from unclassified countries were highly variable due to the very small number of participants.

#### 3.2.2 Comparative analysis: upper-middle vs. lower-middle income countries

**Figure 2:**
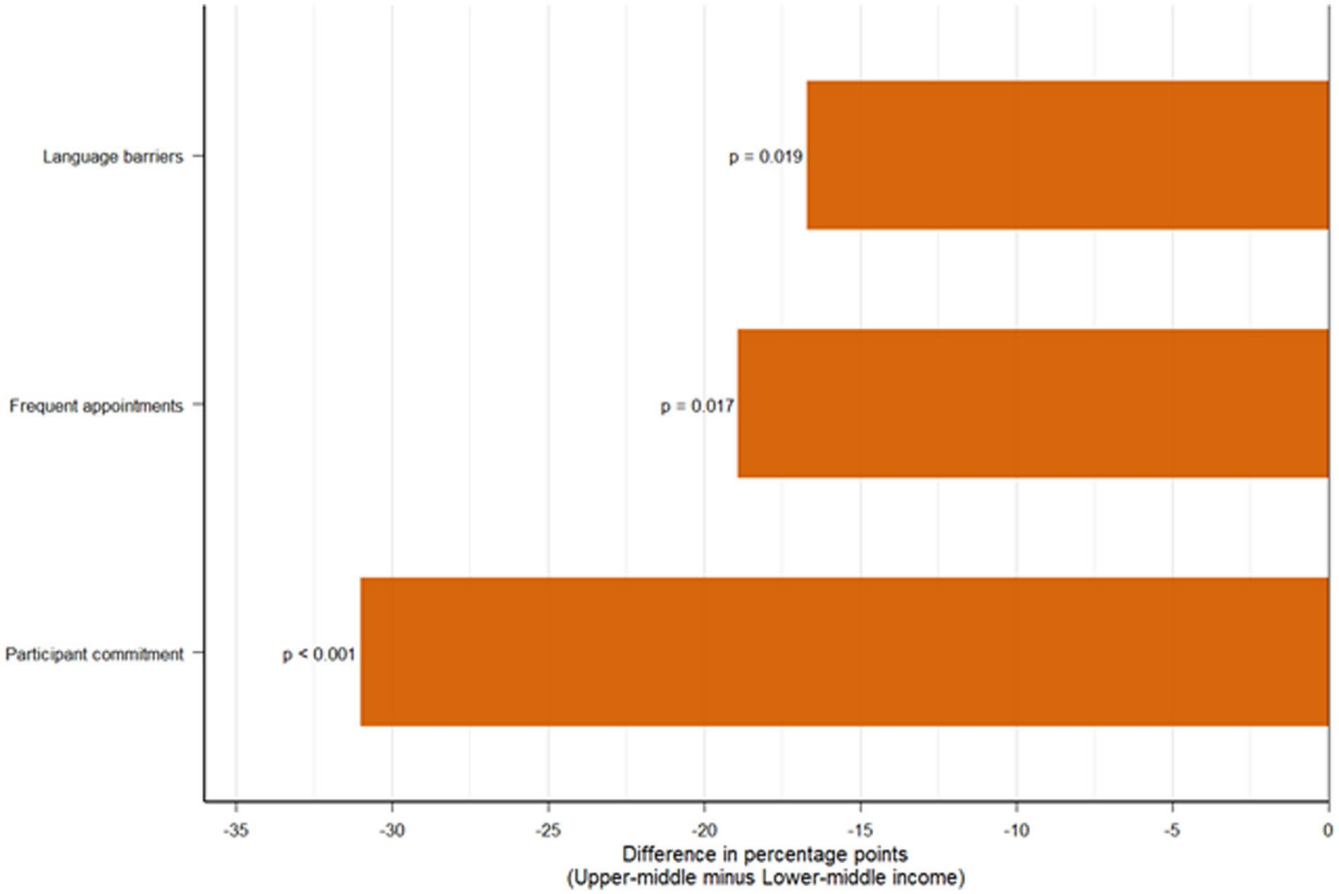
Barriers showing significant differences between upper-middle and lower-middle income countries. Differences in the proportion of respondents rating each barrier as highly important, comparing upper-middle income (*n* = 249) with lower-middle income (*n* = 53) countries. Bars represent the absolute difference in percentage points (upper-middle minus lower-middle). Negative values indicate a higher proportion of lower-middle income respondents rating the barrier as highly important. Only statistically significant differences (*p* < 0.05) are shown: participant commitment (Δ = −31.0 pp, *p* < 0.001), frequent appointments (Δ = −18.9 pp, *p* = 0.017), and language barriers (Δ = −16.7 pp, *p* = 0.019).

Statistical comparisons between upper-middle- and lower-middle-income groups are presented in Table 3 and Figure 2. Of the sixteen barriers assessed, three demonstrated statistically significant differences. Participant commitment showed the most substantial contrast, with markedly higher ratings in lower-middle-income countries (73.6% vs. 42.6%; *p* < 0.001; Cramér’s V = 0.28). Frequent appointments were also more often rated as highly important in lower-middle-income settings (56.6% vs. 37.8%; *p* = 0.020; Cramér’s V = 0.17), suggesting greater logistical burden for participants. In addition, language barriers were more frequently prioritized in lower-middle-income contexts (39.6% vs. 22.9%; *p* = 0.016; Cramér’s V = 0.17). All remaining barriers showed no statistically significant differences between groups, and the three most critical barriers—funding, research materials, and time constraints—displayed virtually identical ratings across settings, indicating that these obstacles are pervasive and universally felt irrespective of country income level.

**Table 3:** Comparison of high-importance barrier ratings between upper-middle and lower-middle income countries.

Barrier	Upper-middle (*n*, %)	Lower-middle (*n*, %)	Δ (pp)	χ^2^ test	Cramér’s V	*p*-value	Significance
Participant commitment	106 (42.6%)	39 (73.6%)	−31.0	χ^2^(1) = 15.5	0.28	<0.001	***
Frequent appointments	94 (37.8%)	30 (56.6%)	−18.9	χ^2^(1) = 5.7	0.17	0.017	*
Language barriers	57 (22.9%)	21 (39.6%)	−16.7	χ^2^(1) = 5.6	0.17	0.019	*
Risks	107 (43.0%)	30 (56.6%)	−13.6	χ^2^(1) = 2.7	0.11	0.097	ns
Inclusion/exclusion criteria	86 (34.5%)	25 (47.2%)	−12.6	χ^2^(1) = 2.5	0.10	0.115	ns
Telemedicine	78 (31.3%)	23 (43.4%)	−12.1	χ^2^(1) = 2.3	0.09	0.126	ns
Additional biopsies	126 (50.6%)	32 (60.4%)	−9.8	χ^2^(1) = 1.3	0.07	0.253	ns
Patient enrollment	105 (42.2%)	26 (49.1%)	−6.9	χ^2^(1) = 0.6	0.05	0.444	ns
Laboratory tests	108 (43.4%)	26 (49.1%)	−5.7	χ^2^(1) = 0.4	0.04	0.546	ns
Trained personnel	149 (59.8%)	34 (64.2%)	−4.3	χ^2^(1) = 0.2	0.03	0.668	ns
Insurance/coverage	128 (51.4%)	25 (47.2%)	+4.2	χ^2^(1) = 0.2	0.03	0.683	ns
Competent authority procedures	154 (61.8%)	31 (58.5%)	+3.4	χ^2^(1) = 0.1	0.02	0.764	ns
Ethics committee procedures	157 (63.1%)	35 (66.0%)	−3.0	χ^2^(1) = 0.06	0.02	0.800	ns
Funding	211 (84.7%)	46 (86.8%)	−2.1	χ^2^(1) = 0.03	0.01	0.866	ns
Time constraints	174 (69.9%)	37 (69.8%)	+0.1	χ^2^(1) < 0.01	<0.01	1.000	ns
Research materials	181 (72.7%)	38 (71.7%)	+1.0	χ^2^(1) < 0.01	<0.01	1.000	ns

Δ (pp): absolute difference in percentage points (lower-middle minus upper-middle). χ^2^(df): Pearson chi-square statistic with degrees of freedom. Effect size reported via Cramér’s V, interpreted as: small (0.10), medium (0.30), large (0.50). Significance codes: ***p < 0.001; **p < 0.01; *p < 0.05; ns = not significant. All *p*-values two-sided.

#### 3.2.3 Sensitivity analysis

To ensure that the findings were not an artifact of dichotomizing the original 3-point Likert scale, Mann–Whitney U tests were conducted on the complete ordinal responses and are summarized in Supplementary Table S1. Results were highly consistent with those of the chi-square analyses: participant commitment remained highly significant (*p* < 0.001), frequent appointments remained significant (*p* = 0.02), and language barriers showed borderline significance (*p* = 0.05). All remaining barriers remained non-significant, confirming that the overall conclusions are robust across analytical approaches.

### 3.3 Predictors of research protocol participation

**Figure 3:**
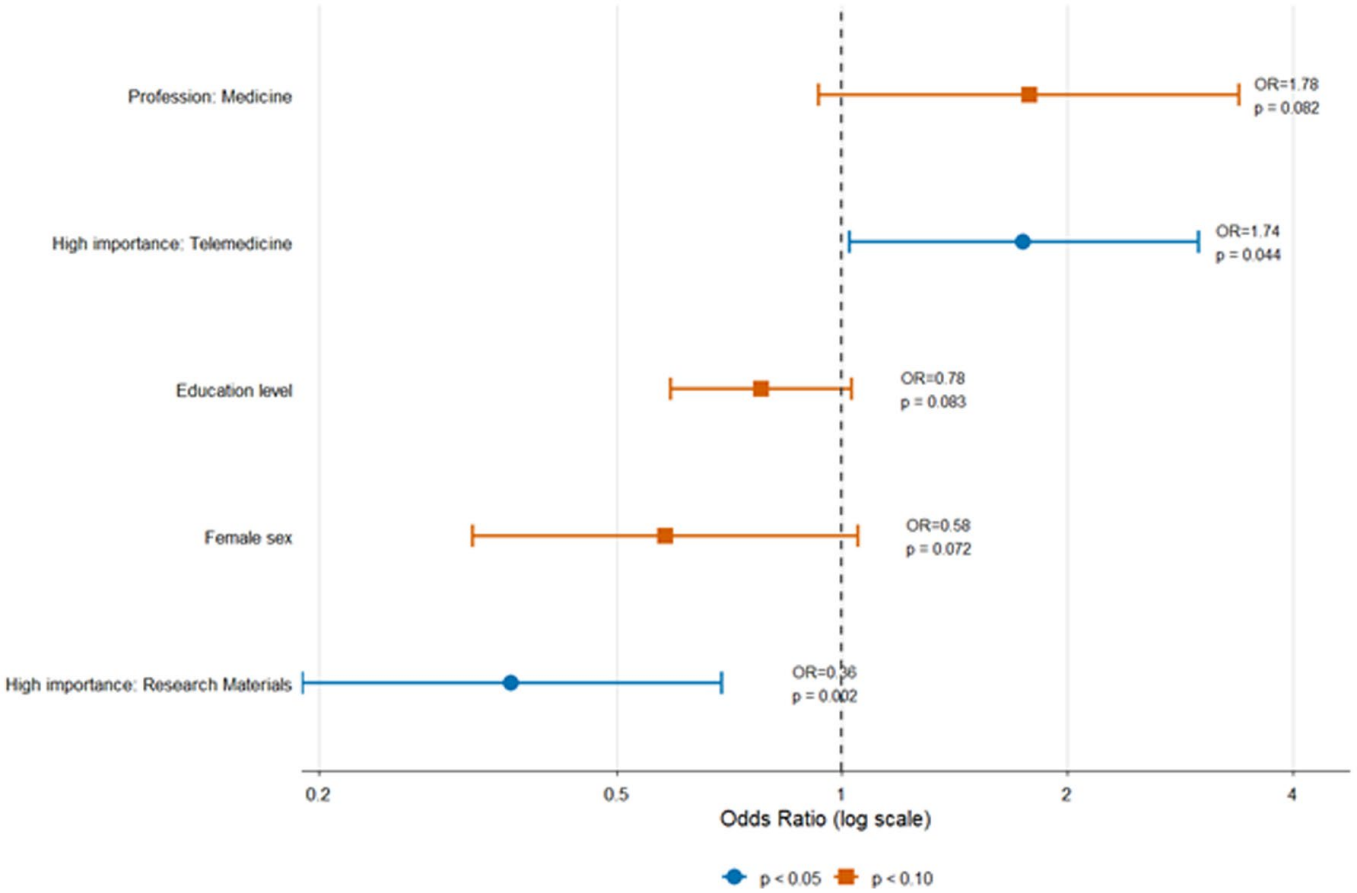
Predictors of research participation in a multivariable logistic regression model. Forest plot showing predictors of research participation in the multivariable logistic regression model (*n* = 302). Points represent adjusted odds ratios (ORs), and horizontal lines indicate 95% confidence intervals. The vertical dashed line denotes the null value (OR = 1). Predictors with *p* < 0.05 are shown in blue, while those with 0.05 ≤ *p* < 0.10 are shown in orange. Significant predictors included high-importance ratings for research materials (OR = 0.36, *p* = 0.002) and telemedicine (OR = 1.74, *p* = 0.044). Marginal associations were observed for profession (medicine), female sex, and education level.

The independent predictors of research participation were examined using a multivariable logistic regression model restricted to the 302 respondents from upper-middle- and lower-middle-income countries. Full model results are shown in Table 4, and effect estimates are visualized in Figure 3. The final model incorporated thirteen predictors, including income classification, sex, age, education level, four professional categories, and five key perceived barriers. Model diagnostics indicated acceptable performance: all variance inflation factors were below 3, ruling out problematic multicollinearity; the Hosmer–Lemeshow goodness-of-fit test demonstrated adequate calibration (χ^2^ = 6.87, df = 8, *p* = 0.549); the area under the ROC curve (AUC = 0.675) suggested acceptable discriminatory ability; and the model explained 6.7% of the variance in research participation (Nagelkerke pseudo-R^2^ = 0.067), with an overall classification accuracy of 65.2%. Although the explained variance was modest, the diagnostic indicators support the reliability and interpretability of the parameter estimates.

**Table 4:** Multivariable logistic regression predicting research participation (*n* = 302).

Variable	β coefficient	OR	95% CI	SE	*p*-value	Significance
Intercept	1.86	6.40	1.28–31.98	0.82	0.024	*
Income (upper-middle)	−0.19	0.82	0.42–1.61	0.34	0.570	ns
Female sex	−0.55	0.58	0.32–1.05	0.31	0.072	†
Age	−0.12	0.89	0.71–1.12	0.12	0.327	ns
Education level	−0.25	0.78	0.59–1.03	0.14	0.083	†
Profession: nursing	−0.21	0.81	0.29–2.23	0.52	0.680	ns
Profession: pharmacy	−0.35	0.71	0.29–1.70	0.45	0.440	ns
Profession: medicine	0.57	1.78	0.93–3.39	0.33	0.082	†
Profession: other	0.49	1.63	0.24–11.15	0.98	0.617	ns
Barrier: funding (high importance)	0.29	1.34	0.62–2.86	0.39	0.455	ns
Barrier: research materials (high importance)	−1.02	0.36	0.19–0.69	0.33	0.002	****
Barrier: time constraints (high importance)	0.27	1.30	0.76–2.23	0.27	0.332	ns
Barrier: participant commitment (high importance)	0.20	1.22	0.72–2.06	0.27	0.457	ns
Barrier: telemedicine (high importance)	0.56	1.74	1.02–2.99	0.28	0.044	*

β, logistic regression coefficient; OR, odds ratio; SE, standard error; CI, confidence interval. Odds ratios were derived from exponentiated β coefficients, and 95% CIs were calculated as exp(β ± 1.96 × SE). *p*-values correspond to two-sided Wald tests. Significance codes indicate: *p < 0.05; **p < 0.01; † 0.05 ≤ *p* < 0.10 (trend-level); ns, not statistically significant. The reference category for professional background was nutrition/dietetics. Model diagnostics are reported in the main text (Hosmer–Lemeshow test, AUC, VIFs, Nagelkerke R^2^).

Two predictors demonstrated statistically significant associations with research participation. Rating research materials as a high-importance barrier was associated with substantially lower odds of participation (OR = 0.36, 95% CI: 0.19–0.69; *p* = 0.002), a counterintuitive result that may reflect greater awareness of material resource limitations among those with prior research experience or, conversely, the resilience required to engage in research despite such constraints. In contrast, rating telemedicine as highly important was associated with greater odds of participation (OR = 1.74, 95% CI: 1.02–2.99; *p* = 0.044), suggesting that individuals actively involved in research may be more attuned to or engaged with emerging digital modalities relevant to clinical and nutritional research.

Several additional predictors approached statistical significance but did not meet conventional thresholds. Female respondents showed a tendency toward lower participation (OR = 0.58, 95% CI: 0.32–1.05; *p* = 0.072), raising the possibility of underlying gender disparities in research engagement. Higher educational attainment displayed an unexpected trend toward lower participation (OR = 0.78, 95% CI: 0.59–1.03; *p* = 0.083), potentially attributable to coding structure or unmeasured confounding factors. Likewise, respondents in medicine showed a trend toward higher participation relative to nutritionists/dietitians, the reference category (OR = 1.78, 95% CI: 0.93–3.39; *p* = 0.082).

Predictors that did not demonstrate significant associations included income classification, age, other professional groups, and perceived importance of funding, time constraints, and patient enrollment (all *p* > 0.10). Their nonsignificance suggests that, within this sample, sociodemographic factors may be less influential than specific operational or attitudinal barriers in shaping engagement in research activities.

### 3.4 Cluster analysis of barrier perception

**Figure 4:**
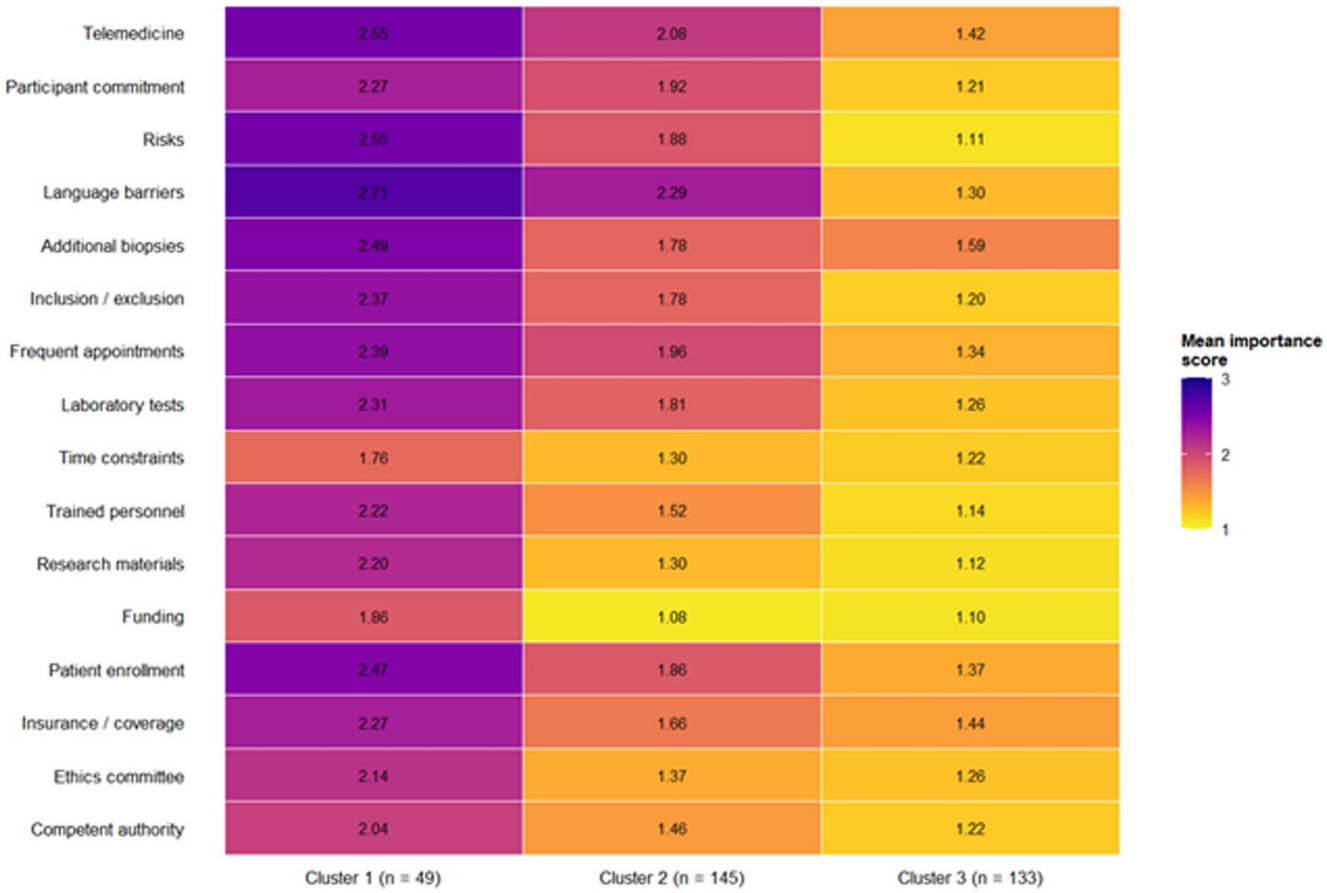
Cluster analysis of mean importance scores for research barriers. Heatmap of mean importance scores (1 = high importance, 2 = medium, 3 = low) for 16 research barriers across three researcher clusters (*n* = 327). Each cell represents the average perceived importance of a barrier within each cluster, with darker shades indicating higher perceived importance (closer to 1). Cluster 1 (*n* = 49) shows overall moderate barrier perception; Cluster 2 (*n* = 145) displays consistently high importance across operational and resource-related barriers; and Cluster 3 (*n* = 133) exhibits uniformly high importance ratings across nearly all barriers. This visualization highlights distinct profiles of barrier perception within the research community.

K-means clustering was applied to identify distinct profiles of researchers based on their perception of all sixteen assessed barriers, with the results summarized in Table 5 and illustrated in Figure 4. The optimal solution consisted of three clusters (k = 3), determined through triangulation of the elbow method—showing stabilization of inertia at k = 3—the highest silhouette coefficient among candidate models (0.21), and conceptual interpretability. The first profile, comprising 49 participants (15.0% of the sample), showed a moderate level of barrier perception. Although this group identified time constraints, funding limitations, competent authority procedures, ethics committee requirements, and research materials as their top concerns, the mean scores for these barriers were closer to 2 (medium importance), indicating that these researchers generally perceived obstacles as less severe. Demographically, Cluster 1 showed balanced representation across professions and income groups, suggesting a heterogeneous but relatively resilient subgroup.

**Table 5:** Mean importance ratings of research barriers across k-means clusters (k = 3).

Barrier	Cluster 1 (moderate barriers) *n* = 49	Cluster 2 (high general barriers) *n* = 145	Cluster 3 (very high specific barriers) *n* = 133
Regulatory barriers			
Competent authority procedures	2.04	1.46	1.22
Ethics committee procedures	2.14	1.37	1.26
Insurance/coverage	2.27	1.66	1.44
Patient enrollment	2.47	1.86	1.37
Resource-related barriers			
Funding	1.86	1.08	1.10
Research materials	2.20	1.30	1.12
Trained personnel	2.22	1.52	1.14
Time constraints	1.76	1.30	1.22
Laboratory tests	2.31	1.81	1.26
Operational barriers			
Frequent appointments	2.39	1.96	1.34
Inclusion/exclusion criteria	2.37	1.78	1.20
Additional biopsies	2.49	1.78	1.59
Communication-related barriers			
Language barriers	2.71	2.29	1.30
Clinical/participant barriers			
Risks	2.55	1.88	1.11
Participant commitment	2.27	1.92	1.21

Lower scores indicate higher perceived barrier importance (1 = high, 2 = medium, 3 = low). Cluster labels are descriptive and based on the interpretation of mean score patterns across 16 barriers. Cluster membership was derived using k-means clustering (k = 3), selected using the elbow criterion, silhouette index (maximum at k = 3), and conceptual interpretability. Full cluster diagnostics and variance explained are reported in the main text. No statistically significant association was observed between cluster membership and country income classification (χ^2^ test, *p* = 0.34).

The second profile, representing 145 participants (44.3%), corresponded to a pattern of high general barriers and constituted the largest cluster. Members of this group consistently rated resource-related obstacles as highly important, with particularly elevated ratings for funding, time constraints, research materials, ethics committee procedures, and regulatory approvals. This profile aligns closely with the overall distribution of the full sample and represents the “typical” researcher facing multiple systemic and operational constraints.

The third profile, consisting of 133 participants (40.7%), exhibited a pattern of very high specific barriers, characterized by exceptionally elevated ratings across nearly all operational and personnel-related obstacles. Funding, participant commitment, research materials, availability of trained personnel, and requirements for additional biopsies all showed mean scores near 1, indicating widespread perception of high severity. This group appears to represent individuals working in particularly challenging research environments or those with heightened sensitivity to the cumulative effect of multiple barriers.

Cluster membership did not differ significantly by country income classification; chi-square testing showed no association between cluster assignment and income group (*p* = 0.34). This finding suggests that differences in barrier perception are not solely determined by macroeconomic context. Still, it may instead reflect institutional factors, local research ecosystems, or individual experience within clinical and nutritional research environments.

### 3.5 Sensitivity analyses

A series of sensitivity analyses was conducted to evaluate the robustness of the main findings across alternative income-group classifications. In the first analysis, the six respondents from unclassified countries (Cuba and Venezuela) were included in the lower-middle-income group, creating an expanded comparison with upper-middle-income countries. As shown in Supplementary Table S1, the results were virtually identical to the primary analysis. Participant commitment remained highly significant (*p* < 0.001), and the pattern of non-significant barriers remained unchanged, indicating that excluding unclassified countries did not materially influence the conclusions.

A second analysis examined the broadest possible contrast by merging upper-middle- and high-income countries into a single group and comparing them against lower-middle-income countries plus unclassified countries. This approach increased the sample size in both comparison groups (*n* = 268 vs. *n* = 59). The results again confirmed the stability of the findings: participant commitment remained significantly different between groups (*p* = 0.001), while frequent appointments and language barriers remained statistically significant. No additional barriers emerged as significant, demonstrating that the observed differences were consistent across alternative grouping strategies.

For completeness, descriptive summaries are also provided for participants from high-income and unclassified countries; however, in accordance with the pre-specified analytic plan and minimum sample size requirements (*n* ≥ 30), no statistical comparisons or hypothesis tests were performed for these groups. Among respondents from high-income countries (*n* = 19), 84.2% were female, and 89.5% reported prior or current participation in research. Their most frequently cited high-importance barriers were time constraints (94.7%), ethics committee procedures (78.9%), and funding (78.9%), whereas patient enrollment was the least cited (26.3%). In unclassified countries (*n* = 6), 33.3% were female, and all participants reported research experience. The most salient barriers in this group were research materials (83.3%), funding (66.7%), and ethics committee procedures (66.7%). Given the very small sample sizes, these descriptive patterns should be interpreted with considerable caution, and no inferences may be drawn regarding differences with other income groups.

## 4 Discussion

This cross-sectional study of 327 healthcare professionals across 19 Latin American and Caribbean countries identified multilevel barriers to clinical and nutritional research and compared perceptions of these barriers between upper-middle-income and lower-middle-income countries. Three key findings emerged from our analysis.

First, funding emerged as the overwhelmingly dominant barrier, cited as highly important by 84.4% of all participants, with remarkably consistent ratings across both income groups (upper-middle: 84.7% vs. lower-middle: 86.8%, *p* = 0.87). This universal acknowledgment of funding limitations transcends country income classifications and suggests that inadequate financial investment in clinical and nutritional research represents a systemic, region-wide challenge. Research materials (71.6%) and time constraints (70.9%) constituted the second tier of critical barriers, also showing no significant variation by income level. These findings align with previous studies documenting pervasive resource limitations as the primary impediment to health research in low- and middle-income countries (21).

Second, lower-middle-income countries face distinct operational challenges related to participant engagement and study logistics. Three barriers showed statistically significant differences: participant commitment to study protocols (73.6% vs. 42.6%, *p* < 0.001), requirements for frequent appointments (56.6% vs. 37.8%, *p* = 0.02), and language barriers (39.6% vs. 22.9%, p = 0.02). These findings suggest that, beyond universal resource constraints, lower-middle-income settings contend with additional patient-level and infrastructure-related obstacles that may reflect limited health system capacity, lower health literacy, transportation difficulties, or language diversity in research protocols not adapted to local contexts. The 31-percentage-point difference in participant commitment represents a substantial operational burden that may disproportionately affect research feasibility in resource-constrained settings (22, 23).

Third, contrary to our hypothesis that income-level differences would be pronounced across many barriers, most barriers showed no significant variation between upper-middle and lower-middle income countries. Regulatory barriers (ethics committees, competent authorities), patient enrollment, insurance issues, and most procedural barriers were perceived as equally challenging, regardless of income classification (24). This unexpected homogeneity suggests that within the middle-income spectrum, structural barriers to research may be driven more by regional or institutional factors than by national income levels (25). It also indicates that the dichotomy between “middle-income” countries may be less meaningful than the broader distinction between high-income and middle/low-income settings, or that within-country heterogeneity exceeds between-country differences (26).

In addition, gender disparities also emerged as a significant barrier. Our results—where being female was associated with lower odds of participation—align with findings reported by Silva et al. and Ozkazanc et al. (27), who documented those systemic inequalities, including limited access to research funding and mentorship, disproportionately restrict female researchers’ engagement in high-quality studies (27, 28). Addressing such disparities necessitates targeted policy interventions that protect research time for female investigators and enhance their access to leadership and collaboration opportunities (29).

The role of telemedicine in clinical research presents both opportunities and challenges. Although telemedicine holds the potential to overcome geographical and logistical barriers, our study found that a higher valuation of telemedicine was paradoxically associated with reduced participation. Ciocca et al. (30) and Glass et al. (31) have discussed how underdeveloped digital infrastructures and limited digital literacy in the region could hamper the effective implementation of telemedicine in research contexts. Consequently, technological investments must accompany capacity-building initiatives to realize the full potential of telemedicine in advancing research.

The discrepancies in patient enrollment between income groups further underscore the impact of socioeconomic disparities. Researchers from lower-middle countries face amplified challenges, such as lower insurance coverage and limited outreach capabilities, which constrain recruitment efforts (32, 33). In addition, factors such as language barriers and stringent laboratory test requirements, although not always statistically significant, indicate underlying systemic inequities that restrict research inclusiveness (34, 35).

Our cluster analysis identified three distinct researcher profiles based on barrier perception. This segmentation suggests that interventions must be customized; for instance, targeted mentoring for clusters facing high overall barriers and capacity-building initiatives for those reporting moderate difficulties. Vicente-Crespo et al. (36) emphasize that tailored interventions informed by institutional capacity analyses can significantly enhance research productivity in lower-middle-income settings. Complementing this view, Balán (37) argues that strengthening institutional support and streamlining administrative processes are critical to creating a supportive research environment.

Finally, our findings underscore the necessity of interdisciplinary and regional collaborations—the work by Carpio et al. (38) and Torres et al. (39) demonstrates that socioeconomic inequality and inconsistent funding policies contribute to an unstable research environment, which can be mitigated by coordinated, region-wide policy initiatives. Shively et al. (40) further advocates for capacity-building programs that are tailored to the unique socioeconomic and institutional contexts of Latin America. Moreover, partnerships among governmental bodies, academic institutions, and international organizations are pivotal in mobilizing adequate resources and harmonizing research protocols, as illustrated by Bonini et al. (41) and García-Cerde et al. (42).

Given these multifaceted challenges, a comprehensive, multilevel approach is required. Our study recommends that future research adopt a mixed-methods design to capture better the dynamic interplay among individual, institutional, and systemic factors that affect research participation. Policymakers should prioritize increased funding, regulatory harmonization, digital infrastructure investments, and targeted interventions to address gender disparities and other structural inequalities. Only through such concerted efforts can clinical and nutritional research in Latin America reach its full potential.

This study has several significant limitations that should be carefully considered when interpreting the results. First, the cross-sectional design precludes causal inferences about the relationships between barriers and research participation, limiting our ability to establish temporal sequences or determine causality. Second, reliance on self-reported data introduces potential recall bias and social desirability bias, which may have influenced participants’ ratings of barrier importance and their reported research experiences. Third, the sampling strategy resulted in an uneven geographic and economic distribution, with 91.1% of participants from medium/low-income countries. This significantly reduced the statistical power for meaningful comparisons across income groups and may limit the generalizability of the findings to high-income Latin American countries. Furthermore, the use of non-probabilistic sampling introduces selection bias and limits generalizability; however, this approach is widely accepted for exploratory research in resource-constrained settings (43). Fourth, the focus on healthcare professionals who had already participated in research likely introduced selection bias, systematically excluding individuals who may have encountered insurmountable barriers to research involvement, thus potentially underestimating the true magnitude of research obstacles. Fifth, the study did not capture institutional-level variables such as university research policies, infrastructure quality, administrative support, or institutional culture, which are known to influence research capacity significantly. Seventh, the survey was conducted during the COVID-19 pandemic (September 2021 to February 2022), which may have temporarily altered research priorities, funding availability, and institutional support, potentially affecting the stability and generalizability of barrier perceptions. Finally, the three-point Likert scale, while facilitating analysis, may have limited the nuanced assessment of barrier importance compared to scales with greater granularity.

Additionally, some references are older than 2019, as recent literature on barriers to clinical and nutritional research in Latin America is scarce. Foundational studies remain relevant due to persistent structural challenges and limited regional research output in this field, as highlighted in recent reviews and research capacity assessments (24, 38).

Future research should employ mixed-methods approaches to explore the interplay among individual, institutional, and systemic barriers to clinical and nutritional research. A longitudinal study tracking changes in research capacity following specific interventions would provide valuable evidence on effective strategies for overcoming barriers.

The cluster analysis findings warrant further investigation to understand how researcher profiles evolve and respond to policy interventions. Implementation science approaches could evaluate the effectiveness of targeted strategies for different research groups. Finally, expanding research to include perspectives from funding agencies, ethics committees, and policymakers would provide a more comprehensive understanding of the research ecosystem.

## 5 Conclusion

This study provides empirical evidence of multilevel barriers to clinical and nutritional research in Latin America, demonstrating that while funding constraints affect all countries regardless of income level, lower-middle-income countries face additional challenges related to participant engagement and study logistics. The methodological rigor of our approach—including transparent income classification, pre-specified sample size thresholds, conservative handling of uncertain cases, and comprehensive sensitivity analyses—establishes a model for honest reporting of limitations in observational research with convenience sampling.

By explicitly distinguishing between descriptive and inferential findings and acknowledging the constraints imposed by sample imbalance, we provide a foundation for future research while avoiding overinterpretation of limited data. The identification of distinct barrier perception profiles suggests that capacity-building interventions must be tailored to local contexts and individual needs rather than adopting one-size-fits-all approaches.

Strengthening clinical and nutritional research capacity in Latin America will require sustained commitment from multiple stakeholders—governments increasing research funding, institutions implementing supportive policies, regulatory bodies streamlining approval processes, and research networks fostering collaboration and mentorship. Only through such comprehensive, coordinated efforts can the region realize its potential to generate evidence-based knowledge that improves health outcomes for its diverse populations.

## Data Availability

The raw data supporting the conclusions of this article will be made available by the authors, without undue reservation.

## Author’s note

The study was conceived, conducted, and originally submitted while JA-I, CR-G, and DS-R were affiliated with Universidad UTE (until 1 October 2025). Peer-review revisions, corrections, and the final version were completed at the Pontificia Universidad Católica del Ecuador (PUCE), Quito, Ecuador.
