# Supplementary material for: Multilevel barriers to clinical and nutritional research in Latin America: a socioeconomic comparative analysis
Source: Front Nutr. 2025 Dec 31;12:1599344. doi: 10.3389/fnut.2025.1599344 (PMC12801517; doi:10.3389/fnut.2025.1599344)
Supplement: Supplementary file 1 [file Table_1.docx]

**Table S1. Geographic and Income Distribution of Study Participants (N = 327)**

| **Country** | **World Bank Income Classification** | **n** | **%** |
| --- | --- | --- | --- |
| Argentina | Upper-middle | 24 | 7.3 |
| Brazil | Upper-middle | 89 | 27.2 |
| Chile | High | 1 | 0.3 |
| Colombia | Upper-middle | 19 | 5.8 |
| Costa Rica | Upper-middle | 1 | 0.3 |
| Cuba | Unclassified | 5 | 1.5 |
| Ecuador | Upper-middle | 36 | 11.0 |
| El Salvador | Lower-middle | 7 | 2.1 |
| Spain | High | 1 | 0.3 |
| United States | High | 4 | 1.2 |
| Guatemala | Lower-middle | 37 | 11.3 |
| Mexico | Upper-middle | 46 | 14.1 |
| Nicaragua | Lower-middle | 1 | 0.3 |
| Panama | High | 9 | 2.8 |
| Peru | Upper-middle | 21 | 6.4 |
| Dominican Republic | Upper-middle | 13 | 4.0 |
| Uruguay | High | 4 | 1.2 |
| Venezuela | Unclassified | 1 | 0.3 |
| Paraguay | Lower-middle | 8 | 2.4 |
| **Total** |  | **327** | **100** |

Table S1. Geographic and income distribution of study participants by country and World Bank income classification (N = 327). Data are presented as absolute number (n) and percentage (%). Countries are listed alphabetically. World Bank income classifications based on 2022 GNI per capita thresholds.
