# Supplementary figures and images for: Multilevel barriers to clinical and nutritional research in Latin America: a socioeconomic comparative analysis
Source: Front Nutr. 2025 Dec 31;12:1599344. doi: 10.3389/fnut.2025.1599344 (PMC12801517; doi:10.3389/fnut.2025.1599344)

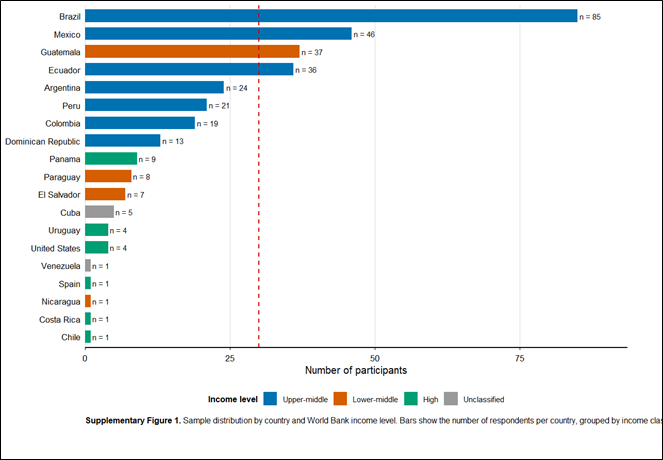

Supplement: Supplementary file 2 [file Image_1.JPEG]
